# LONG-TERM FUNCTIONAL AND CLINICAL OUTCOME OF COMBINED TARGETED MUSCLE REINNERVATION AND OSSEOINTEGRATION FOR FUNCTIONAL BIONIC RECONSTRUCTION IN TRANSHUMERAL AMPUTEES: A CASE SERIES

**DOI:** 10.2340/jrm.v56.34141

**Published:** 2024-05-21

**Authors:** Agnes STURMA, Anna BOESENDORFER, Clemens GSTOETTNER, Benedikt BAUMGARTNER, Stefan SALMINGER, Dario FARINA, Rickard BRÅNEMARK, Ivan VUJAKLIJA, Gerhard M. HOBUSCH, Oskar C. ASZMANN

**Affiliations:** 1 Degree Program Physiotherapy, Department of Health Sciences, University of Applied Sciences FH Campus Vienna, Vienna; 2 Clinical Laboratory for Bionic Extremity Reconstruction, Department of Plastic, Reconstructive and Aesthetic Surgery, Medical University of Vienna, Vienna; 3 Department of Plastic, Reconstructive and Aesthetic Surgery, Medical University of Vienna, Vienna; 4 AUVA Trauma Hospital Lorenz Böhler–European Hand Trauma Center, Vienna, Austria; 5 Department of Bioengineering, Imperial College London, London, UK; 6 Department of Orthopaedics, Institute of Clinical Sciences, Sahlgrenska Academy, University of Gothenburg, Gothenburg, Sweden; 7 K. Lisa Yang Center for Bionics, MIT Media Lab, Massachusetts Institute of Technology, Cambridge, MA, USA; 8 Department of Electrical Engineering and Automation, Aalto University, Espoo, Finland; 9 Department of Orthopedics and Trauma Surgery, Medical University of Vienna, Vienna, Austria

**Keywords:** amputation, function, osseointegration, bone-anchored prosthesis, targeted muscle reinnervation, transhumeral, upper extremity, case series

## Abstract

**Objective:**

To describe and evaluate the combination of osseointegration and nerve transfers in 3 transhumeral amputees.

**Design:**

Case series.

**Patients:**

Three male patients with a unilateral traumatic transhumeral amputation.

**Methods:**

Patients received a combination of osseointegration and targeted muscle reinnervation surgery. Rehabilitation included graded weight training, range of motion exercises, biofeedback, table-top prosthesis training, and controlling the actual device. The impairment in daily life, health-related quality of life, and pain before and after the intervention was evaluated in these patients. Their shoulder range of motion, prosthesis embodiment, and function were documented at a 2- to 5-year follow-up.

**Results:**

All 3 patients attended rehabilitation and used their myoelectric prosthesis on a daily basis. Two patients had full shoulder range of motion with the prosthesis, while the other patient had 55° of abduction and 45° of anteversion. They became more independent in their daily life activities after the intervention and incorporated their prosthesis into their body scheme to a high extent.

**Conclusion:**

These results indicate that patients can benefit from the combined procedure. However, the patients’ perspective, risks of the surgical procedures, and the relatively long rehabilitation procedure need to be incorporated in the decision-making.

LAY ABSTRACT

Shoulder and elbow amputees use their prosthesis primarily for grasping and manipulating objects. A common type of prostheses uses the activity of the muscles of the affected limb for controlling it. These devices are called “myoelectric prostheses”. Unfortunately, due to anatomical and technical restrictions, they do not allow for a natural control and are often perceived as uncomfortable and heavy. Nerve transfers can be used to overcome anatomical constraints and improve prosthesis control. Furthermore, comfort and embodiment can be promoted by the use of bone anchoring: metal implants inserted in the bone, through the skin, providing direct firm attachment of the device. We evaluated the first ever 3 patients who had received a combination of both of these procedures. The analysis showed this approach to be viable, resulting in improved patient-reported upper limb function in daily life and a sense that the prosthesis is part of their body.

Proximal amputations of the upper limb pose a great challenge to reconstructive surgeons and orthopaedic technicians alike. Useful prosthetic reconstruction requires reliable, high-bandwidth information transfer between body and machine, as well as mechanically stable anchorage of the device (1). Conventional prosthetic fittings at the transhumeral level inevitably have a few major drawbacks: that of rotational instability and restricted mobility of the shoulder joint, as well as impairments of the contralateral side due to the extensive sockets and belts required (2–4). Additionally, limited myosignals require switching strategies (such as co-contraction, double impulses, 4-channel control) to address multiple joint movements, which results in unintuitive, slow, and cumbersome device control (5–7). In combination, these limitations lead to low satisfaction and high abandonment rates in upper limb prosthesis users (4, 8–10), factors that are inevitably associated with unemployment and low quality of life. The acceptance rate of proximal prosthesis users differs in the literature, with some authors mentioning 43% (8) or 50% (10), and others describing a primary rejection rate of 10.4% and secondary rejection rate of 27.5% in proximal amputations (9). Improvements to the functional and mechanical interface are therefore especially important for this high-demanding group of patients.

In recent years, 2 clinical innovations were introduced with the aim of improving both of these issues: targeted muscle reinnervation (TMR) and osseointegration (OI). In TMR (11), residual nerves are rerouted to alternative muscles (in transhumeral amputees, usually short head of biceps, brachialis, lateral head of triceps, and brachioradialis) thus generating a greater number of biosignals, which in turn allows for direct control of a larger number of prosthesis functions. TMR is associated with a reduction of phantom limb pain and improved functional outcomes compared with conventional 2-electrode myoelectric prostheses. While the risk profile is low, prosthetic rehabilitation takes about a year (12, 13). OI aims to improve prosthetic attachment through direct anchorage of the device to a percutaneous bone implant in the residual limb. A percutaneous titanium implant is inserted into the humus to enable a direct attachment and stable human–prosthesis connection along with a better range of motion of the shoulder (2). In a retrospective study with 18 transhumeral amputees, the following risks of OI were reported: 15 superficial infections, 8 skin reactions, 8 incomplete fractures at first surgery, 3 defective bony canals at secondary surgery, 3 avascular skin flap necroses, 3 implant failures as well as 1 deep implant infection (14). The complications seem to be dependent on the experience of the medical team.

While patient-reported and functional results of each procedure alone, or in combination with implanted components in experimental settings, have been reported previously (2, 13, 15, 16), we propose that the combination of both is feasible and enables good prosthetic function in daily life.

Thus, the aim of this study is to describe a first case series of 3 patients with an amputation at a transhumeral level who received the combination of TMR and OI, including their medical history, rehabilitation methods applied, and objective functional and patient-reported outcomes measures (PROMs). The latter can serve as a first set of baseline data describing the clinical reality in this specific group of people with high upper limb amputation.

## METHODS

### General procedure

**Fig. 1:**
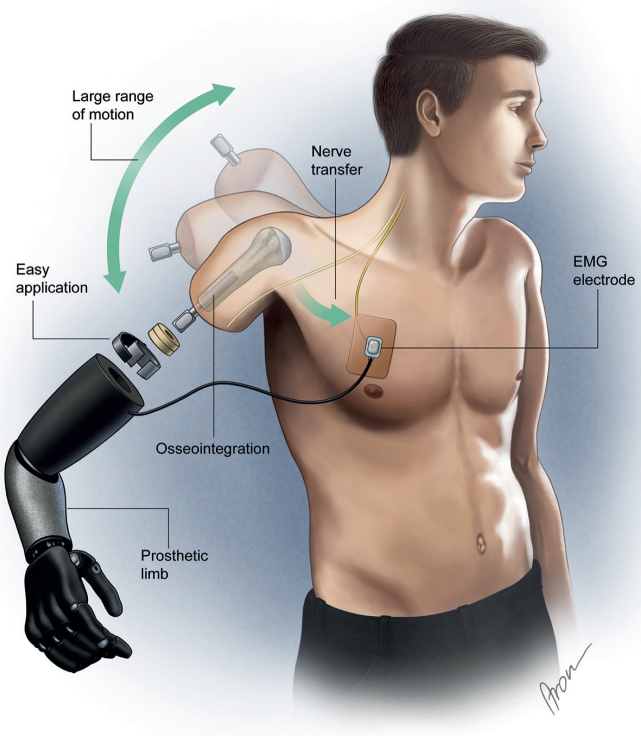
Prosthetic components in a transhumeral amputee after receiving targeted muscle reinnervation and osseointegration. EMG: electromyographic.

Three transhumeral amputees have been recruited and have received combined surgery of bone anchoring and TMR at our centre. In all of them, the Osseointegrated Prostheses for the Rehabilitation of Amputees (OPRA) implant system (Integrum AB, Mölndal, Sweden), is used. This system consists of 2 main components: a threaded and coated titanium fixture, which is implanted into the humerus, and a skin-penetrating abutment, secured to the fixture by the abutment screw, where the prosthesis can be mounted directly. The attached prosthesis is controlled via muscle signals from naturally innervated residual muscles at the stump. TMR describes the surgical rerouting of nerves serving the now amputated hand to muscles of the residual limb. The procedure aims to multiply the available muscle sites by selectively transferring major nerves in the residual limb to separated muscle heads, creating new signals for prosthetic control. Thus, intuitive prosthetic control is achieved. The number of nerve transfers and available individually controllable muscle sites depends on the anatomical prerequisites of the patient. The combined concept is presented in Fig. 1.

**Fig. 2:**
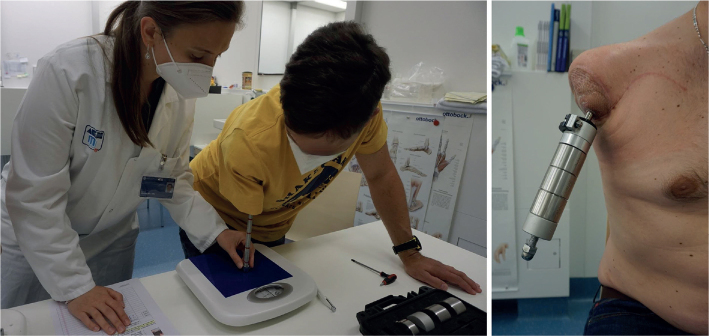
Axial loading with weight scale (left) and weight prosthesis training (right) for rehabilitation of osseointegration.

Subsequent rehabilitation was performed by an occupational therapist (OT) and/or physiotherapist (PT). For TMR rehabilitation, the procedure as described by Sturma at al. (12, 18) was followed. *Interventions covered:* standard postsurgical interventions (oedema and pain control, scar treatment), endurance training, improving range of motion and body symmetry, activation of sensory-motor cortex (mirror therapy, imaginary movements, lateralization training), MyoTesting and MyoTraining with surface electromyographic (sEMG) biofeedback, table-top prosthesis training and prosthesis training, as well as clinical follow-up examinations. Graded weight training and axial loading as needed after OI (see Fig. 2) was applied according to Jönsson et al. (2). Each patient received a training prosthesis with the weight increased weekly until the weight of the final prosthesis was reached. The weight added per week (50 g versus 100 g) depended on the patient’s bone quality/length as assessed via X-ray. Additionally, axial loading with increasing pressure on a weight scale was administered, and abutment cleaning was instructed. The number of therapy sessions was planned according to the availability (family duties, work, and education) and needs of the patients, also taking into account their travel time (ranging from 2.5 to 5.5 hours per direction) (18).

### Patients

**Fig. 3:**
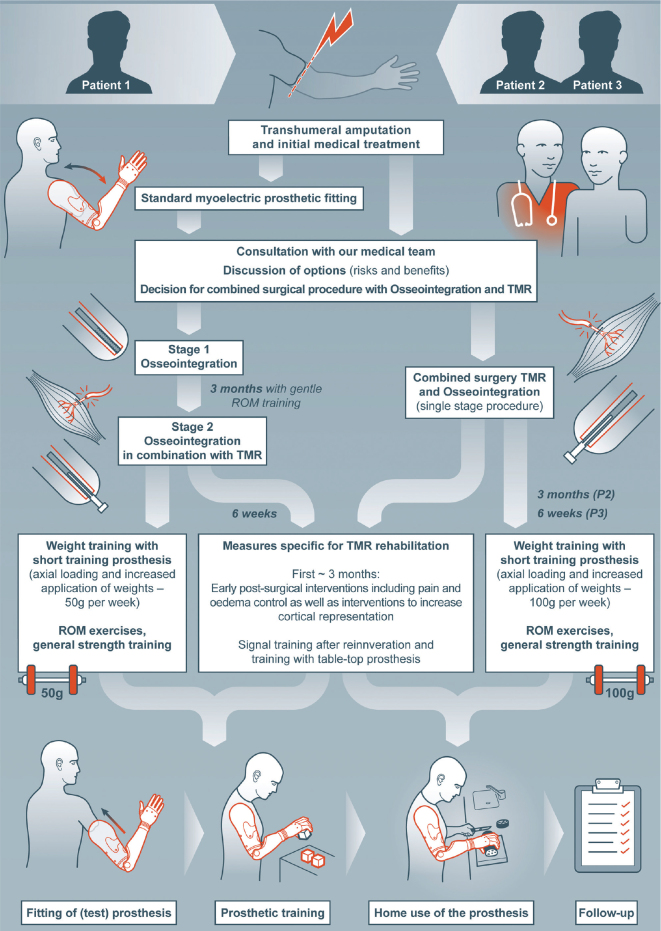
Rehabilitation process of each patient. TMR: targeted muscle reinnervation; ROM: range of motion; P2: patient 2; P3: patient 3.

Three male patients were included in this case series. They consulted our centre for bionic extremity reconstruction between 2018 and 2021 (see Tables I and II for their characteristics and details on the medical treatment) with the wish for an improvement in extremity function. All had a unilateral traumatic upper limb amputation at the transhumeral level and inclusion criteria for OI/TMR were evaluated (2, 12, 18). Assessment criteria included dissatisfaction with conventional socket prosthesis if already fitted, short residual bone, wish/expression for best possible fitting, willingness/high motivation to participate in a relatively long rehabilitation process, funding (provided by health insurance), wish for active lifestyle, and clear understanding of risks and possible benefits after patient information provided by the team. Exclusion criteria were low bone quality (e.g. former exposure to radiation), untreated psychological comorbidities, lack of compliance, and/or language comprehension. The aim of the combined surgery was improved functionality and independence in daily life. The patients’ individual rehabilitation process is summarized in Fig. 3.

**Table I:** Patient characteristics

Patient	Gender	Age at amputation	Amputation level	Side of amputation (dominant/non-dominant)	Cause of amputation	Time from OI+TMR surgery to assessment
1	Male	36 years	Short TH (appr. 6 cm*)	Right (dominant)	Work accident	2 years
2	Male	17 years	TH	Left (non-dominant)	Traffic accident	2 years
3	Male	52 years	TH	Left (non-dominant)	Work accident	5 years

OI: osseointegration; TH: transhumeral; TMR: targeted muscle reinnervation.

* Full bone length, from the subchondral plate of the head of the humerus to the distal end of the bone.

**Table II:** Surgery, rehabilitation and prosthesis details

Patient	Time from amputation to OI+TMR surgery	OI surgery	TMR surgery	OI rehabilitation	Prosthesis
1	29 months (to stage I)	Stage 1: 3/2021 Stage 2: 6/2021 OPRA System	6/2021: – Median nerve → pectoralis major muscle (abdominal part) – Radial nerve/posterior cord → latissimus dorsi muscle	Slow protocol	Ottobock Dynamic Arm Plus, Electric Wrist Rotator, Vincent Hand 4 (2 electrodes)
2	6 months	Single stage 2/2020 OPRA System	2/2020 – Ulnar nerve → biceps brachii muscle (short head) – Median nerve → brachialis muscle – Radial nerve (deep branch) → brachioradialis muscle and triceps muscle (lateral head)	Fast protocol	Ottobock Dynamic Arm Plus, Electric Wrist Rotator, Vincent Hand 4 (4 electrodes)
3	10 months	Single stage 5/2018 OPRA System	5/2018 – Median nerve → brachialis muscle – Ulnar nerve → biceps brachii muscle (short head) – Radial nerve (deep branch) → brachioradialis muscle and triceps muscle (lateral head)	Fast protocol	Ottobock Dynamic Arm Plus, Electric Wrist Rotator, Sensor Hand Speed (4 electrodes)

OI: osseointegration; OPRA: Osseointegrated Prostheses for the Rehabilitation of Amputees; TMR: targeted muscle reinnervation.

Data collection was approved by the ethical review board of the Medical University of Vienna. All patients gave written informed consent for their study participation.

**Fig. 4:**
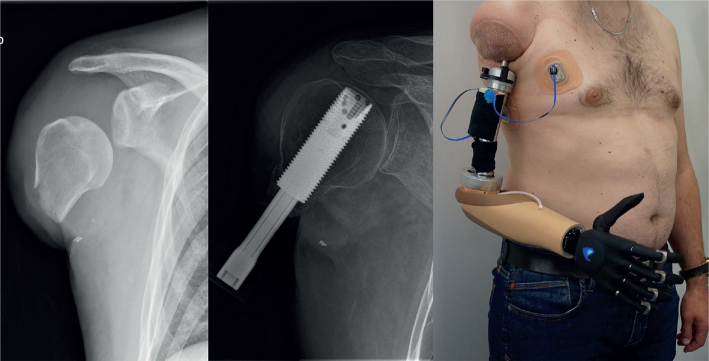
AP X-ray of the remaining humerus bone before targeted muscle reinnervation and osseointegration surgery (left), AP X-ray 16 weeks after stage 2 surgery (middle) and after prosthetic fitting with adhesive electrodes in patient 1 (right).

*Patient 1* (36 years, male) had a work accident (agricultural worker) in October 2018, which resulted in a transhumeral amputation with a very short humerus (approximately 6 cm total bone length, from the subchondral plate of the head of the humerus to the distal end of the bone) as depicted in Fig. 4. He was already fitted with a conventional glenohumeral myoelectric socket prosthesis with a Dynamic Arm Plus and Sensor Hand Speed (Ottobock Healthcare Products GmbH, Duderstadt, Germany). He used it on a daily basis (from morning to evening). Control was achieved through 2 electrodes on the chest and the remaining deltoid. To control all joints of the prosthesis, co-contraction was used as a switching mechanism. For stable attachment of the prosthesis a harness to the contralateral chest and a shoulder cap were necessary, which resulted in complete loss of shoulder mobility. He was restricted in daily task activities (such as dressing and cutting food) and needed the help of others. The main indication for OI was expected improved shoulder mobility after the procedure. At the time of presentation in our centre, he suffered from phantom limb pain (VAS 65/100) despite his pain medication, which included pregabalin, tetrahydrocannabinol and opioids (Lyrica^®^, Dronabiol^®^, Hydal^®^, and Fentanyl^®^). In March 2021, stage I surgery for OI took place. Three months later, OI Stage II and TMR surgery were performed (nerve transfer matrix for each patient can be found in Table II). The nerve transfers were needed to obtain separate signals independent of shoulder movements. Due to the short humerus, the team decided on a slow rehabilitation protocol for weight loading. In the first quarter of 2022, the patient was fitted with a Dynamic Arm Plus, Electric Wrist Rotator (Ottobock Healthcare Products GmbH, Duderstadt, Germany) and Vincent Hand 4 (Vincent Systems GmbH, Karlsruhe, Germany) with 2 adhesive electrodes (on pectoralis major and latissimus dorsi). Patient 1 used adhesive electrodes, which provides a reliable interface for his 2 myosignals (see Fig. 4). However, these need to be changed every 3 to 5 days, as well as for personal hygiene. With co-contraction, he had full use of the prosthesis joints. Over the course of rehabilitation, the patient attended 13 outpatient therapy sessions and 5 multidisciplinary consultations at our centre. He received additional therapy during the post-surgical hospital stay and intensive inpatient rehabilitation at another centre over a period of 4 weeks. Additionally, follow-ups via emails, online meetings, and phone calls were performed. At the time of the follow-up visit, the patient was already working full time as an independent farmer again.

**Fig. 5:**
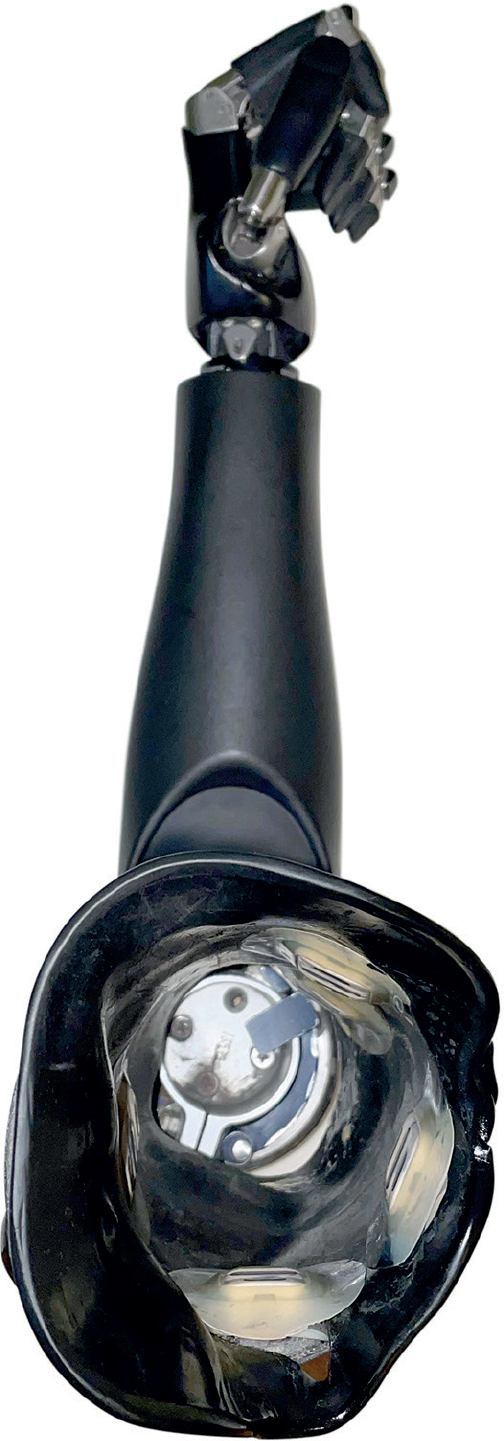
Osseointegrated socket prosthesis with four active electrodes (and one inactive electrode) in patient 2.

*Patient 2* (17 years, male) had a traffic accident in August 2019, which resulted in a transhumeral amputation. At first patient consultation, the team decided on a combination of TMR and OI due to an active lifestyle, young age, full range of motion of the shoulder joint, and personal need for a high-end fitting. The patient was instructed to train shoulder mobility, muscle strength, and imagine movements of the lost arm. He was not fitted with a prosthesis during the waiting time for surgery. In February 2020, a one-stage OI and TMR surgery was performed followed by post-surgical therapy interventions. He was fitted with a Dynamic Arm Plus, Electric Wrist Rotator (Ottobock Healthcare Products GmbH, Duderstadt, Germany) and a Vincent Hand 4 (Vincent Systems GmbH, Karlsruhe, Germany), all directly controlled with 4 electrodes. He used a socket housing his 4 active electrodes (see Fig. 5), which is connected to the prosthesis via a cable. He could directly control open/close of the hand (TMR sites lateral head of triceps and short head of biceps) and elbow flexion/extension (original innervation of long head of triceps and biceps). For using the rotation unit, a switching mode (co-contraction) was needed. To use all grips of the multiarticulating Vincent Hand 4, further switching modes were needed. In total, 17 outpatient therapy sessions and 2 multidisciplinary consultations took place at our centre in combination with follow-ups via email and phone. The patient already showed reliable control over a table-top prosthesis 6 months after surgery and was subsequently fitted with an end device. The actual prosthetic fitting was delayed for over a year due to late approval by the health insurance company. At the time of follow-up assessments, the patient was at the end of his professional school education (as a trainee) with the outlook of working in an office job.

*Patient 3* (52 years, male) had a work accident in July 2017 resulting in a transhumeral amputation. He was not fitted with a conventional socket prosthesis when he visited our centre. The indications for the combined surgery were neuroma resection for TMR, volume fluctuation on the stump for OI, as well as possible full range of motion of the shoulder joint. In May 2018, his single-stage OI and TMR surgery took place. After successful post-surgical rehabilitation, the patient was fitted with a Dynamic Arm Plus, Electric Wrist Rotator and Sensor Hand Speed with 4 electrodes (Ottobock Healthcare Products GmbH, Duderstadt, Germany) for prosthesis control. He also used a socket for his 4 electrodes (similar to patient 2; TMR sites lateral head of triceps and short head of biceps, original innervation of long head of triceps and biceps) and used co-contraction as switching mode. Twelve outpatient therapy sessions and 9 multidisciplinary consultations were conducted over the course of rehabilitation. Additionally, the patient attended 3 intensive inpatient rehabilitation stays at another centre focusing on general health promotion and some prosthetic training (10 weeks, 4 weeks, and 4 weeks). During the follow-up period, 2 soft tissue corrections due to weight changes were required. Patient 3 was in premature retirement as he could not return to his physically high-demanding work as a foreman.

### Assessments

The patient-reported questionnaire “Disabilities of the Arm, Shoulder and Hand” (DASH) was used as the primary outcome, with scores collected before surgery (with conventional prosthesis in patient 1, and no prostheses in patients 2 and 3) and after completing rehabilitation. Within 30 items (such as turn a key, prepare a meal, weakness in arm, shoulder, or hand), a disability score in daily life can be calculated (0 = no disability, 100 = highest possible disability) (19, 20).

For secondary outcomes, a battery of assessments was used. Range of motion of the shoulder in frontal and sagittal plane was recorded. Patients were asked to mark their current pain level on a visual analogue scale and note pain medications. Health-related quality of life was assessed using the self-reported SF-36 Health survey (SF-36), 4-week recall. Physical (PCS) and mental component summary scales (MSC) were calculated and presented in comparison with norm data (0 = worst quality of life, 100 = best quality of life) (21, 22). To evaluate prosthesis embodiment, 6 questions were rated by the patients on a Likert scale from 0 (never) to 10 (always): “*I had the feeling that the prosthesis was part of my body”*; *“I felt the prosthesis only as a tool, and not as a part of my body*”; “*I did bimanual tasks with my intact arm/hand together with my prosthesis*”; “*I felt that I had full control over the prosthesis*”; “*I liked wearing the prosthesis*”, and “*I felt that my prosthesis looked like a real part of the body*” (23). Hand function was evaluated with the Action Research Arm Test (ARAT) (24) and the Southampton Hand Assessment Procedure (SHAP) test (25). Both tests include grasping tasks (such as grasping a ball, moving and releasing it) and tasks related to daily activities (e.g. pouring water from one object to another). Fifty-seven points of the ARAT and an index of function of 100 points of the SHAP test relates to normal hand function (0 = no hand function). The Clothespin Relocation Test (CPRT) was used to evaluate the ability to use different degrees of freedom of the prosthesis (26). The patients were asked to move 3 clothespins from a horizontal to a vertical bar and the time was measured. After 3 repetitions, a mean time was calculated and presented in seconds. In a study of Kyberd, Hussaini, and Maillet, non-amputated individuals needed a mean time of 4.08 s for this task (27). In addition to these quantitative assessments, patients were asked to describe the effects of the intervention in their daily life.

This battery of tests was designed with the idea of providing a holistic set of assessment tools and baseline data for this unique patient population. All assessments were instructed by the same OT and/or PT. Disability in daily life, pain, and quality of life were evaluated before surgery and at the end of rehabilitation, while prosthetic-related functional tests (range of motion with the prosthesis as well as ARAT, SHAP, and CPRT) were used only at the end of rehabilitation with the final fitting of the prosthesis. The embodiment questionnaire was administered in patient 1 before and after intervention, and only after the intervention in patients 2 and 3 (who had no prosthesis beforehand). The post-rehabilitation assessments were performed in March 2023 (patient 1), June 2022 (patient 2), and August 2023 (patient 3).

### Data analysis

Quantitative data for each patient are presented and descriptive statistics (mean and standard deviation [SD]) were applied, using SPSS 27 (IBM Corp, Armonk, NY, USA).

## RESULTS

### Patient-reported outcomes

All 3 patients improved their DASH scores (primary outcome) before surgery and post-rehabilitation assessment (2–5 years after surgery). The score of patient 1 changed from 45.8 (with conventional myoelectrical prosthesis) to 23.3, in patient 2 from 20 (no prosthesis) to 10.8, and in patient 3 from 57.1 (no fitting) to 37.5 (see Table III). Individual domains can be seen in Table SI. Patient 1 had a subjective great pre-post difference in “stiffness of shoulder” (question 28), which improved from extreme to none. Also, there were relevant improvements in 6 bimanual daily activities (such as “open tight/new jar”, “prepare a meal”, “change a lightbulb overhead”). “Interference with normal social activities” (question 22) improved from moderately to not at all. Only the “wash your back” activity was downgraded from mild to severe difficulty, and was in fact performed without a prosthesis. Patient 2 had good/low disability scores even before the intervention; however, better performance of at least 2 points was seen in “garden/hard work”, “use a knife to cut food”, and “recreational activities with some force or impact through arm, shoulder or hand”. Patient 3 reported an improvement in “stiffness of shoulder/arm” as well, and that he “felt more capable/confident/useful” (question 30). A decrease in disability (of at least 2 points) in 10 activities, such as “open tight/new jar”, “turn key”, “push open a heavy door”, “prepare a meal”, “change a lightbulb overhead”, and “use a knife to cut food” were documented.

**Table III:** Results of patient-reported outcome measures before the interventions and at follow-up

Patient	DASH		PCS		MCS		VAS	
	Pre	Post	Pre	Post	Pre	Post	Pre	Post
1	45.8	23.3	34	32	53	58	65	30
2	20	10.8	54	61	65	57	0	0
3	57.1	37.5	34	32	34	62	25	46
Mean	41	23.9	40.7	41.7	50.7	59	30	25.3
SD	19	13.4	11.5	16.7	15.6	2.6	32.8	23.4

DASH: Disabilities of the Arm, Shoulder and Hand; PCS: physical component summary scale of the SF-36; MCS: mental component summary scale of the SF-36; VAS: visual analogue scale of pain; SD: standard deviation. Lower DASH and VAS scores indicate a better result (min. 0, max. 100), whereas higher PSC and MSC scores indicate a better quality of life (min. 0, max. 100).

PCS scores of SF-36 decreased between pre and post from 34 to 32 in 2 patients (1 and 3) and increased from 54 to 61 in patient 2. MSC scores of SF-36 improved from 53 to 58 (patient 1), 34 to 62 (patient 3), and reduced from 65 to 57 (patient 2). Individual answers on the SF-36 can be seen in Table SI. Pain level ameliorated from 65 to 30 (on a visual analogue scale) in patient 1 and a reduction of pain medication was possible. While patient 2 had no pain at any point in time, pain was aggravated from 25 to 46 (on a visual analogue scale) in patient 3, but without the need to change any pain medication. The raw data of the individual answers to the questionnaire can be obtained from Table SI.

All 3 patients used the OI prosthesis on a daily basis (6–15 h) and reported high embodiment of the device. The perception that the prosthesis was part of their body was rated at 8 (in patients 1 and 2) and 10 ( = always) in patient 3. The detailed embodiment scores are displayed in Table IV.

**Table IV:** Wearing time of prosthesis and embodiment questions

Patient	1		2	3
	pre	post	post	post
Use time of myoelectric prosthesis	Daily, morning to evening, approx. 12 h/daily	Daily, several hours but not continuously, approx. 6 h/daily	Daily, morning to evening, approx. 15 h/daily	Daily, several hours but not continuously, approx. 12 h/daily
“I had the feeling that the prosthesis was part of my body”	6	8	8	10
“I felt the prosthesis only as a tool, and not as a part of my body”	9	2	1	9
“I did bimanual tasks with my intact arm/hand together with my prosthesis”	9	9	8	6
“I felt that I had full control over the prosthesis”	9	8	9	8
“I liked wearing the prosthesis”	9	10	8	9
“I felt that my prosthesis looked like a real part of the body”	1	5	5	7

0: never; 10: always.

### Subjective changes in daily life

When asked about how their activities of daily life changed with the procedure, patient 1 reported that he was now able to perform different bilateral tasks of daily life independently (e.g. cutting his food), which he could not do with his conventional prosthesis. Patient 2, who did not have a fitting before inclusion, pointed out the social importance of his prosthesis and that he enjoyed that others would not realise immediately that he had lost an arm. Patient 3 had a mechanical prosthesis as an additional fitting, which he would use for potentially dangerous mechanical work, such as operating a chainsaw.

### Functional outcome measures

**Fig. 6:**
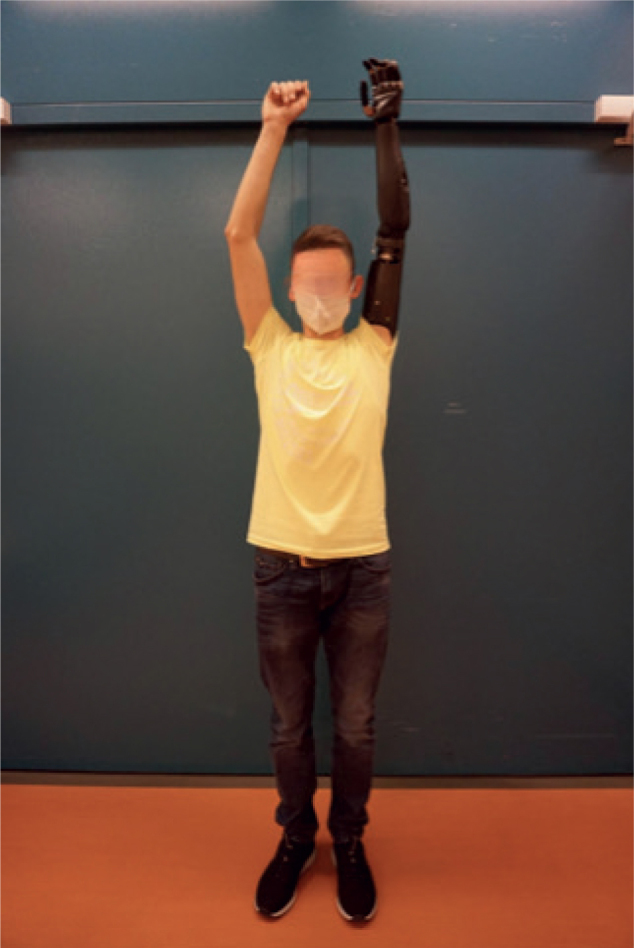
Full range of motion in shoulder abduction while wearing the prosthesis in patient 2.

Analysing the functional results for each patient (patients 1 to 3), the SHAP index of function scores was 30, 29, and 31 (max. of 100, which would represent normal hand function) (see Table V). ARAT scores were 31, 30, and 24 (max. 57) and times for CPRT were 51.39 s, 47.41 s, and 52.25 s. In addition, patients 2 and 3 presented with full range of motion of the shoulder while wearing the prosthesis (see Fig. 6 and Video S1), whereas patient 1 showed 55° of shoulder abduction and 45° of anteversion. Without the prosthesis, patient 1 could move his shoulder in 80° of abduction and 105° of anteversion. The raw data on the individual tasks of the functional assessments can be obtained from Table SI.

**Table V:** Results of functional outcome measures and shoulder range of motion at follow-up

Patient	SHAP	ARAT	CPRT	Shoulder ABD with prosthesis (frontal)	Shoulder AV with prosthesis (sagittal)
1	30	31	51.39s	55°	45°
2	29	30	47.41s	180°	180°
3	31	24	52.25s	180°	180°
Mean	30	28.3	50.35s	138.3°	135°
SD	1	3.8	2.58s	72.2°	78°

SHAP: Southampton Hand Assessment Procedure (min. 0, max. 100); ARAT: Action Research Arm Test (min. 0, max. 57); CPRT: Clothespin Relocation Test; ABD: abduction; AV: anteversion; s: seconds, SD: standard deviation.

## DISCUSSION

Three transhumeral amputees received a combination of OI and TMR surgery to improve prosthetic attachment and control. While patient 1 had previously been fitted with a conventional socket, the others had not used any prosthetic device before our interventions. After surgery, all patients were fitted with myoelectrical prostheses according to their specific requirements. After completing the rehabilitation protocol, they reported daily prosthesis use and showed lower impairment in daily life.

Patient 1 had a very short humerus, which is why he was fitted with a prosthetic socket enclosing the shoulder joint prior to the intervention. He had no mobility of the shoulder joint (0° of abduction and anteversion) with the previous socket and was limited in many daily life activities. OI was therefore the only way to benefit from his existing shoulder mobility and to provide a transhumeral fitting. With the OI fitting, he achieved 55° of abduction and 45° of anteversion in the shoulder. This limitation is likely due to the loss of deltoid function in such a short stump, with the rotator cuff remaining as the main driver for abduction. Based on the findings of Ricardo et al. (28), it can be expected, however, that the ROM might further improve. Nonetheless, even with the current ROM, the patient perceived a great improvement of shoulder function as reflected in his low rating of “stiffness of shoulder” in the DASH questionnaire. This enabled him to dress himself, cut his food, and eat independently. With these improvements in mind, we would therefore conclude that OI and TMR are attractive options for patients with a short humerus. The bone length of 6 cm, however, seems to be a reasonable minimum.

Aiming for better functionality, supporting the active and independent lifestyle of patient 2, and high amount of manual work as well as varying residual limb volume due to weight changes in patient 3 were the main reasons for the decision to undergo the combined surgery of OI and TMR. These different motivations for undergoing the procedure were also reflected in the patients’ focus when reporting changes in activities of daily living after the intervention. While patient 1 reported great improvements in self-care, including being able to dress himself for the first time since amputation, patient 2 focused on the perceived benefits in social interactions, and patient 3 described work-related tasks, where he carefully chose whether to use his myoelectric prosthesis or the mechanical device.

All patients in our study reported reduced impairment as quantified with the mean [SD] DASH scores at 41 [19] before intervention and 23.9 [13.4] at follow-up. These findings are in line with a case report of a patient receiving OI in combination with a hybrid prosthetic system (electrical elbow and body-powered hook) (28). His DASH score improved from 54 to 17 points. The DASH score is a generic questionnaire used for musculoskeletal disorders of the upper limb. While it is not specific for amputations, the questionnaire is widely used internationally, available in many different languages, and was already previously used in amputee populations (28–31), which enables comparison. Other studies with major unilateral upper limb amputees reported a mean DASH score ranging from 32.8 (30) to 39 (29). While this indicates that our patients perceive less impairment than these patients, little information on the prosthetic fitting in the beforementioned study makes a precise interpretation difficult.

Patients in our study wore their prosthesis on a daily basis (6–15 h), which is similar to the 10–12 h/day described in Ricardo et al. (28) for a 7-year follow up after OI. These long prosthesis wearing times can be considered an indicator of prosthetic comfort and perceived usefulness. It needs to be noted, however, that prosthetic wearing time decreased in patient 1, while he was still wearing his prosthesis on a daily basis. He explained this by the fact that shortly before assessment he had the impression of overuse in the shoulder joint and thus temporarily decreased wearing time. As this may be due to the limited remaining muscular structures with such a short stump, we see this as another indication that his residual bone length of 6 cm is a reasonable minimum. Furthermore, we expect the prosthesis wearing time to increase over time, as noted in Ricardo et al. (28).

In terms of objective functional outcomes in our patients compared with other studies, the picture is inconclusive, which may be caused by a lack of normative data in the field. In a functional outcome study, conventional prosthesis users (34 transhumeral and 5 shoulder disarticulation) achieved a mean [SD] index of function of 11.6 [14.8] in the SHAP, which is lower compared with our results (see Table V) (32). Comparing our functional scores with a cohort (*n* = 10) of TMR patients without OI treated in our clinic (13), the mean [SD] SHAP scores were higher at 40.5 [8.1] and CPRT time was shorter at 34.3 s [14.4 s] for these patients without OI. In the meantime, our patients with a combination of TMR and OI had better ARAT scores (28.3 [3.8] vs 20.4 [1.9]). Thus, based on these outcomes, the benefit of prosthesis function of adding OI is inconclusive. However, the better scores in the SHAP and CPRT in the cohort without OI were probably due to a longer follow-up period and therefore longer home use of the devices. Furthermore, larger studies need to investigate the value of the aforementioned tests in displaying functional benefits and limitations in these specific cohorts.

Another outcome parameter discussed in the literature for OI is the embodiment of the prosthesis. For this, Lundberg et al. (33) interviewed OI users (for lower and upper limb) using a qualitative approach. They all perceived OI as a revolutionary change compared with their previous socket prosthesis. For some patients, the bone anchorage supported the feeling that the prosthesis was part of their body and that they felt “whole” again. Similarly, our patients reported a high level of prosthesis embodiment. Comparing pre and post data of patient 1, who used a prosthesis before our intervention, also led to the conclusion that the intervention indeed increased embodiment of the prosthesis.

While all these outcomes are promising, possible risks of OI also need to be considered in clinical decision-making. They include skin reactions and superficial infection at the skin penetration site and incomplete fracture at the first surgery (14). Loosening and deep implant infections are rather rare in the upper extremity. The standard surgical risks apply to TMR, otherwise there is a low risk profile. Nevertheless, those involved must be aware that high costs are required to finance the surgery, rehabilitation, and cost-intensive prosthetic treatment. A further challenge often described in TMR patients is picking up the multiple biosignals with surface electrodes. In our study, 2 different methods were applied for attaching the electrodes to the stump (adhesive electrodes vs in a socket). The number of electrodes also differed in the patients (2 vs 4). More TMR sites support more intuitive and natural control of the prosthesis by the patients, as for example the ulnar nerve can be used for closing the hand, the musculocutaneous nerve for elbow flexion, and the median nerve for pronation.

Given the invasiveness and possible risks of the procedures, patient education is of utmost importance for decision-making. It should include realistic risks and benefits of the procedure, possible contraindications, and organizational conditions, ranging from costs and timelines to the services the medical team can offer. In addition to the not yet streamlined rehabilitation process for OI and TMR in general, we recommend that only multidisciplinary teams with experience in prosthetic care offer the procedure, to ensure satisfactory outcomes of these complex procedures.

The small number of participants and heterogeneity of the group regarding different surgical approaches and end devices are limitations of this study. We included only male patients (of different ages), which is not uncommon, as more men than women are affected by major amputations of the upper extremity (37). While the prosthetic history of patient 1 (assessed with conventional socket fitting) made his experience hard to compare with patients 2 and 3 (no prosthesis at all), both scenarios can be expected in clinical reality. Another limitation is that functional tests and prosthesis embodiment (in patients 2 and 3) were only assessed at the time of follow-up and no comparison data exist for these patients. Furthermore, patients who would opt for such invasive procedures are not probably comparable to the general population (selection bias).

Despite these limitations and the small number of patients, the data represent high added value for the scientific and clinical community, as there is currently no structured analysis of the outcomes in the investigated group of patients. We have made mention of this beneficial relationship in a case report previously by observing a highly specific bilateral amputee (17). However, to our knowledge this is the first report of 3 unilateral transhumeral amputees where we have systematically investigated functional outcomes, quality of life, embodiment, and pain scores, as well as influences on the rehabilitation process and final outcomes in daily life. More research with higher patient numbers and comparison of the outcomes with different cohorts (TMR and OI, only TMR, only OI, in comparison with conventional socket prostheses etc.) will be helpful in the future.

In conclusion, combining TMR and OI provides a viable option for individuals with transhumeral amputations seeking high-level prosthetic reconstruction. Patients with short residual limbs can benefit most from this procedure. While they would need a “glenohumeral” socket fitting impeding any shoulder range of motion due to the needed stability, OI offers the possibility of maintaining a functional shoulder joint. If these patients still wish to use a myoelectric prosthesis, TMR might be needed to provide sufficient myoelectric signals for prosthetic control. Amputees with longer residual limbs may still benefit from improved ROM due to OI, while having the benefit of a sufficient number of available muscles for several nerve transfers to further improve prosthetic control. For both groups, the rehabilitation protocol needs to be tailored to the specifics of the required surgical procedures, leading to promising functional patient-reported and objective results.

## Supplementary Material

LONG-TERM FUNCTIONAL AND CLINICAL OUTCOME OF COMBINED TARGETED MUSCLE REINNERVATION AND OSSEOINTEGRATION FOR FUNCTIONAL BIONIC RECONSTRUCTION IN TRANSHUMERAL AMPUTEES: A CASE SERIES

LONG-TERM FUNCTIONAL AND CLINICAL OUTCOME OF COMBINED TARGETED MUSCLE REINNERVATION AND OSSEOINTEGRATION FOR FUNCTIONAL BIONIC RECONSTRUCTION IN TRANSHUMERAL AMPUTEES: A CASE SERIES
