# Supplementary material for: LONG-TERM FUNCTIONAL AND CLINICAL OUTCOME OF COMBINED TARGETED MUSCLE REINNERVATION AND OSSEOINTEGRATION FOR FUNCTIONAL BIONIC RECONSTRUCTION IN TRANSHUMERAL AMPUTEES: A CASE SERIES
Source: J Rehabil Med. 2024 May 21;56:34141. doi: 10.2340/jrm.v56.34141 (PMC11135336; doi:10.2340/jrm.v56.34141)
Supplement: LONG-TERM FUNCTIONAL AND CLINICAL OUTCOME OF COMBINED TARGETED MUSCLE REINNERVATION AND OSSEOINTEGRATION FOR FUNCTIONAL BIONIC RECONSTRUCTION IN TRANSHUMERAL AMPUTEES: A CASE SERIES [file JRM-56-34141-s1.pdf]

Supplementary material has been published as submitted. It has not been copyedited, or typeset by Journal of Rehabilitation Medicine

Table SI – Raw data of functional assessments (ARAT, SHAP), standardized questionnaires (DASH, SF-36) and subjective changes in daily life

1. **Raw Data follow-up of Action Research Arm Test (ARAT)**

Tasks ARAT	Patient 1	Patient 2	Patient 3
Grasp subscale			
Block, 10 cm^3	2	2	0
Block, 2.5 cm^3	2	2	2
Block, 5 cm^3	2	2	2
Block, 7.5 cm^3	2	2	2
Cricket ball	2	2	2
Sharpening stone	2	2	2
Grip subscale			
Pour water from one glass to another	2	2	1
Displace 2.25-cm alloy tube from one side of the table to the other	2	2	2
Displace 1-cm alloy tube from one side of the table to the other	2	2	2
Push washer over bolt	2	2	0
Pinch subscale			
Ball bearing, held between ring finger and thumb	0	0	0
Marble, held between index finger and thumb	2	2	2
Marble, held between middle finger and thumb	0	0	0
Marble, held between ring finger and thumb	0	0	0
Ball bearing, held between index finger and thumb	2	2	0

Ball bearing, held between middle finger and thumb	0	0	0
Gross movement subscale			
Hand to behind the neck	1	2	2
Hand to top of head	3	2	3
Hand to mouth	3	2	2
**Total (0 = minimum score; 57 = maximum score)**	**31**	**30**	**24**

Scoring: 3 = normal performance; 2 = task completed, but with great difficulty or abnormally slowly; 1 = task partially completed; 0 = not able to complete any part of the task within 60 seconds

2. **Raw Data follow-up of the Southampton Hand Assessment Procedure (SHAP)**

Tasks SHAP	Patient 1	Patient 2	Patient 3
Abstract Objects			
Light Sphere	10.00	8.84	7.37
Light Tripod	17.94	9.31	12.44
Light Power	8.40	7.28	22.28
Light Lateral	15.41	9.62	44.59
Light Tip	11.31	8.57	14.51
Light Extension	11.63	9.00	7.15
Heavy Sphere	8.56	11.31	73.82
Heavy Tripod	9.12	10.63	18.03
Heavy Power	5.72	8.25	22.73
Heavy Lateral	11.87	10.00	45.26

Heavy Tip	6.46	9.81	7.32
Heavy Extension	8.53	11.72	5.94
Activities of Daily Living (ADLs)			
Pick Up Coins	79.06	100.00	42.79
Button Board	45.93	100.00	36.17
Simulated Food Cutting	50.36	35.66	100.00
Page Turning	13.53	29.28	17.15
Jar Lid	5.19	7.06	12.97
Glass Jug Pouring	43.84	45.40	100.00
Carton Pouring	29.44	23.40	100.00
Lifting a Heavy Object	18.84	1.71	7.81
Lifting a Light Object	9.69	7.40	5.05
Lifting a Tray	7.09	8.44	9.00
Rotate Key	10.85	30.31	100.00
Open/Close Zip	12.88	18.09	100.00
Rotate A Screw	22.87	62.00	100.00
Door Handle	5.19	3.78	2.81
**Index of Function Score (0 = minimum; 100 = maximum score)**	**30**	**29**	**31**

The time for completing each of the task was measured (in seconds). If a task could not be completed or lasted longer than 100.00 seconds, a time of 100.00 was documented.

The index of function was calculated on the website (http://www.shap.ecs.soton.ac.uk/entry.php).

3. **Raw Data pre and follow-up of the Disabilities of the Arm, Shoulder and Hand Questionnaire (DASH)**

Questions DASH	Patient 1		Patient 2		Patient 3	
	pre	post	pre	post	pre	post
Open a tight or new jar.	4	2	3	2	4	1
Write.	2	2	1	1	2	1
Turn a key.	1	1	1	1	3	1
Prepare a meal.	3	1	3	2	5	2
Push open a heavy door.	2	1	1	1	3	1
Place an object on a shelf above your head.	1	1	1	2	3	3
Do heavy household chores (e.g., wash walls, wash floors).	4	3	2	2	4	4
Garden or do yard work.	3	2	3	1	5	3
Make a bed.	3	1	3	2	5	3
Carry a shopping bag or briefcase.	1	1	1	1	1	2
Carry a heavy object (over 10 lbs).	2	1	1	1	2	3
Change a lightbulb overhead.	4	1	1	1	5	3
Wash or blow dry your hair.	1	1	1	1	4	2
Wash your back.	2	4	1	1	5	5
Put on a pullover sweater.	4	2	1	1	2	2
Use a knife to cut food.	3	2	4	2	5	3
Recreational activities which require little effort (e.g., cardplaying, knitting, etc.).	3	1	3	2	5	3
Recreational activities in which you take some force or impact through your arm, shoulder or hand (e.g., golf, hammering, tennis, etc.).	5	4	5	2	4	4

Recreational activities in which you move your arm freely (e.g., playing frisbee, badminton, etc.).	2	3	3	2	4	3
Manage transportation needs (getting from one place to another).	1	1	1	1	1	2
Sexual activities.	2	1	1	1	3	3
During the past week, to what extent has your arm, shoulder or hand problem interfered with your normal social activities with family, friends, neighbours or groups?	3	1	1	2	X	3
During the past week, were you limited in your work or other regular daily activities as a result of your arm, shoulder or hand problem?	2	3	1	2	X	3
Please rate the arm, shoulder or hand pain.	4	3	2	2	2	3
Please rate the arm, shoulder or hand pain when you performed any specific activity.	4	3	1	1	2	3
Please rate the tingling (pins and needles) in your arm, shoulder or hand.	4	3	3	2	3	3
Please rate the weakness in your arm, shoulder or hand.	4	3	1	1	2	2
Please rate the stiffness in your arm, shoulder or hand.	5	1	1	1	3	1
During the past week, how much difficulty have you had sleeping because of the pain in your arm, shoulder or hand?	2	1	1	1	2	2
I feel less capable, less confident or less useful because of my arm, shoulder or hand problem.	4	4	2	1	3	1
**Total (0 = no disability; 100 = maximum disability)**	**45,8**	**23,3**	**20**	**10,8**	**57,1**	**37,5**

Scoring: 1 = no difficulty/not at all; 2 = mild difficulty/slightly; 3 = moderate difficulty/moderately; 4 = severe difficulty/quite a bit; 5 = unable/extremely; X= missing; light green= improvement of 2 points; dark green= improvement of 3 and 4 points; light blue= worsening of 2 points

The total score as reported in the main manuscript is calculated in the following way: sum of all responses, divided by the number of responses, subtracted by one, and then multiplied by 25.

4. Raw Data pre and follow-up of the Short Form 36 Health Survey (SF-36)

Questions SF-36	Patient 1		Patient 2		Patient 3	
	pre	post	pre	post	pre	post
In general, would you say your health is:	Good	Good	Very good	Excellent	Good	Excellent
Compared to one year ago, how would you rate your health in general now	Somewhat better than one year ago	Somewhat better than one year ago	Somewhat worse than one year ago	Somewhat better than one year ago	Much worse now than one year ago	About the same as one year ago
The following questions are about activities you might do during a typical day. Does your health now limit you in these activities? If so, how much?						
Vigorous activities, such as running, lifting heavy objects, participating in strenuous sports.	Yes, limited a lot	Yes, limited a lot	No, not limited at all	No, not limited at all	Yes, limited a lot	Yes, limited a lot
Moderate activities, such as moving a table, pushing a vacuum cleaner, bowling or playing golf	Yes, limited a lot	Yes, limited a little	Yes, limited a little	No, not limited at all	Yes, limited a lot	Yes, limited a lot
Lifting or carrying groceries	Yes, limited a little	No, not limited at all	No, not limited at all	No, not limited at all	Yes, limited a little	Yes, limited a lot
Climbing several flights of stairs	Yes, limited a	No, not	No, not	No, not	Yes, limited a	Yes, limited a

	little	limited at all	limited at all	limited at all	little	little
Climbing one flight of stairs	No, not limited at all	No, not limited at all	No, not limited at all	No, not limited at all	No, not limited at all	No, not limited at all
Bending, kneeling or stooping	Yes, limited a little	No, not limited at all	No, not limited at all	No, not limited at all	Yes, limited a little	Yes, limited a little
Walking more than a mile	Yes, limited a lot	Yes, limited a lot	No, not limited at all	No, not limited at all	No, not limited at all	Yes, limited a little
Walking half a mile	Yes, limited a little	Yes, limited a little	No, not limited at all	No, not limited at all	Yes, limited a little	Yes, limited a little
Walking one hundred yards	No, not limited at all	No, not limited at all	No, not limited at all	No, not limited at all	Yes, limited a little	No, not limited at all
Bathing or dressing yourself	Yes, limited a little	Yes, limited a little	No, not limited at all	No, not limited at all	Yes, limited a lot	Yes, limited a little
During the past 4 weeks, have you had any of the following problems with your work or other regular daily activities as						

a result of your physical health?						
Cut down on the amount of time you spent on work or other activities	No	Yes	No	No	Yes	Yes
Accomplished less than you would like	No	Yes	No	No	Yes	Yes
Were limited in the kind of work or other activities	No	Yes	Yes	No	Yes	Yes
Had difficulty performing the work or other activities (for example, it took extra effort)	No	Yes	No	No	Yes	Yes
During the past 4 weeks, have you had any of the following problems with your work or other regular daily activities as a result of any emotional problems (such as feeling depressed or anxious)?						
Cut down on the amount of time you spent on work or other activities	No	No	No	No	Yes	No
Accomplished less than you would like	No	No	No	No	Yes	No
Didn't do work or other activities as carefully as usual	No	No	No	No	Yes	No
During the past 4 weeks, to what extent has your physical health or emotional problems interfered with your normal social activities with family, friends, neighbours or groups?	Slightly	Slightly	Not at all	Not at all	Moderately	Moderately
How much bodily pain have you had during the past 4 weeks?	Very severe	Moderate	Very mild	None	Moderate	Moderate
During the past 4 weeks, how much did pain interfere with your normal work (including both work outside the	Extremely	A little bit	Not at all	Not at all	Moderately	Moderately

home and housework)?						
These questions are about how you feel and how things have been with you during the past 4 weeks.<br>For each question please give the one answer that comes closest to the way you have been feeling.<br>How much of the time during the past 4 weeks…						
Did you feel full of life?	A little of the time	A good bit of the time	All of the time	A good bit of the time	Some of the time	A good bit of the time
Have you been a very nervous person?	A little of the time	Some of the time	None of the time	None of the time	Some of the time	A little of the time
Have you felt so down in the dumps that nothing could cheer you up?	None of the time	None of the time	None of the time	None of the time	Some of the time	A little of the time
Have you felt calm and peaceful?	Most of the time	Most of the time	All of the time	Most of the time	A good bit of the time	Most of the time
Did you have a lot of energy?	A little of the time	Most of the time	All of the time	Most of the time	Some of the time	All of the time
Have you felt downhearted and low?	Some of the time	A little of the time	None of the time	None of the time	Some of the time	A little of the time
Did you feel worn out?	Most of the time	A good bit of the time	None of the time	A little of the time	Some of the time	Some of the time
Have you been a happy person?	Most of the time	Most of the time	All of the time	Most of the time	Some of the time	Most of the time

Did you feel tired?	All of the time	A good bit of the time	A little of the time	Some of the time	A good bit of the time	A little of the time
During the past 4 weeks, how much of the time has your physical health or emotional problems interfered with your social activities (like visiting friends, relatives, etc.)?	A little of the time	A little of the time	None of the time	None of the time	Some of the time	A little of the time
How TRUE or FALSE is each of the following statements to you?						
I seem to get ill more easily than other people	Definitely false	Definitely false	Definitely false	Definitely false	Don't know	Definitely false
I am as healthy as anybody I know	Mostly true	Mostly false	Definitely true	Definitely true	Mostly false	Definitely true
I expect my health to get worse	Definitely false	Don't know	Definitely false	Definitely false	Don't know	Definitely false
My health is excellent	Mostly true	Mostly true	Definitely true	Definitely true	Don't know	Definitely true
**Physical Component Summary (0 = minimum; 100 = maximum score)**	**34**	**32**	**54**	**61**	**34**	**32**
**Mental Component Summary (0 = minimum; 100 = maximum score)**	**53**	**58**	**65**	**57**	**34**	**62**

The scores as reported in the main manuscript (raw values of physical component summary, mental component summary) are calculated on the website (https://www.hogrefe-online.com/HTSEnvironment/main). A total score of 0 to 100 is possible, higher scores indicate better health-related quality of life.

5. **Subjective changes in daily life**

When asked about how their activities of daily life changed with the procedure, patient 1 reported that he was now able to put on a jacket without help from others, as well as cutting his food, eating soup with a spoon, opening food packages, applying toothpaste while holding the toothbrush with the prosthesis, and in general performing bilateral activities. His youngest child, who was born after the accident, would not shy away to use the prosthesis to hold his hand. On the other hand, the older children would always take his healthy arm. Due to lack of personal benefit, for driving his big agricultural machines (e.g., tractor) he did not wear the myoelectrical prosthesis, only the training prothesis for muscle strengthening.

In a similar way, also patient 3 reported that he would not wear his myoelectric prosthesis for potentially dangerous mechanical work, such as operating a chainsaw. He was fitted with a second mechanical prosthesis that he would use in these cases.

Patient 2 pointed out the social importance of his prosthesis. He was wearing it full day and also during (office) work, and put his focus less on the functional gain, but rather that he enjoyed that others would not realise immediately that he had lost an arm. His previous hobby “flying with model aeroplanes” was still not possible due to insufficient fine motor and coordination skills.
